# Distributed network organization underlying feeding behavior in the mollusk *Lymnaea*

**DOI:** 10.1186/2042-1001-2-4

**Published:** 2012-04-17

**Authors:** Paul R Benjamin

**Affiliations:** 1 School of Life Sciences, University of Sussex, Falmer, Brighton BN1 9QG, UK

**Keywords:** Distributed organization, *Lymnaea*, Molluscan feeding behavior, Networks, Neurons

## Abstract

The aim of the work reviewed here is to relate the properties of individual neurons to network organization and behavior using the feeding system of the gastropod mollusk, *Lymnaea*. Food ingestion in this animal involves sequences of rhythmic biting movements that are initiated by the application of a chemical food stimulus to the lips and esophagus. We investigated how individual neurons contribute to various network functions that are required for the generation of feeding behavior such as rhythm generation, initiation ('decision making'), modulation and hunger and satiety. The data support the view that feeding behavior is generated by a distributed type of network organization with individual neurons often contributing to more than one network function, sharing roles with other neurons. Multitasking in a distributed type of network would be 'economically' sensible in the *Lymnaea *feeding system where only about 100 neurons are available to carry out a variety of complex tasks performed by millions of neurons in the vertebrate nervous system. Having complementary and potentially alternative mechanisms for network functions would also add robustness to what is a 'noisy' network where variable firing rates and synaptic strengths are commonly encountered in electrophysiological recording experiments.

## Introduction

**Figure 1:**
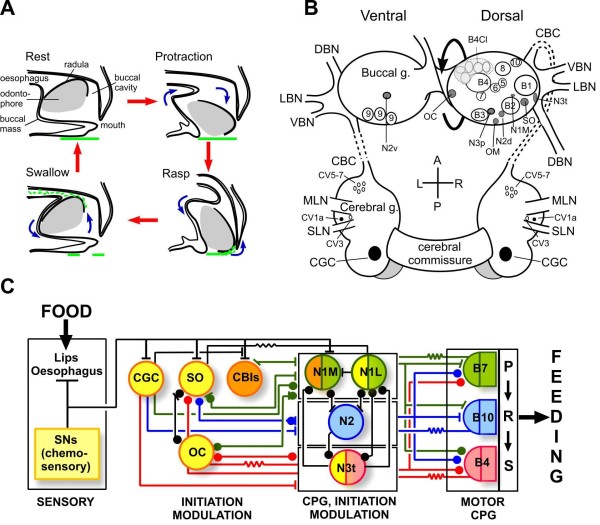
**Movements, neurons and network organization underlying feeding in *Lymnaea***. **(A) **There are four phases in the feeding ingestion cycle. During the protraction phase the buccal mass and radular rotate forwards, the mouth opens and by the end of this phase the radular is pressed on the food substrate. During rasp the radular begins to rotate backwards and scoops the food into the buccal cavity. During swallow the mouth closes and the radular continues to rotate backwards to push the food into the esophagus. The rest phase is a period of inactivity between feeding cycles. In fast rhythms such as the one shown in Figure 2A the rest period is reduced to zero. **(B) **Map of feeding neurons in the buccal ganglia (buccal g.) and cerebral ganglia (cerebral g.). There are symmetrical sets of neurons on left and right side except for the SO that is a single cell that can be either on the left or right side. Unshaded neurons are motoneurons (B1 to B10, CV3, C5 to C7). Shaded neurons are CPG interneurons (N1M, N1L, N2d, N2v, N3p and N3t), modulatory interneurons (OC, SO and CGCs), initiating neurons (CV1a) and sensory neurons (OM). CV1a is part of a larger population of CBIs and the complete map of their locations is shown in Figure 3A. A = anterior; CBC = cerebrobuccal connective; L = left; MLN = median lip nerve; P = posterior; R = right, SLN = superior lip nerve. **(C) **Synaptic connectivity and functions of neurons in the feeding circuit. Modulatory function is indicated by yellow and initiating function by orange. CPG interneurons and motoneurons active during the three phases of the feeding rhythm are indicated by green (P = protraction), blue (R = rasp) and red (S = swallow). Neurons labeled with two colors have two functions. Dots indicate inhibitory chemical synapses, bars excitatory chemical synapses and resistor symbols electrotonic (electrical) synapses. This figure emphasizes the point that many of the neurons have more than function in the feeding network. See Abbreviations for all definitions of neuron types.

Work on rhythmic motor behaviors in invertebrates, such as locomotion, eating and heartbeat, has been extremely valuable in providing general insights into how the nervous system generates behavior [1,2]. An example of a rhythmically active motor network that has made significant contributions to this field is the feeding system of the pond snail, *Lymnaea*. An advantage of this system is that the various mechanisms that are important in generating and controlling rhythmic motor behaviors such as pattern generation, initiation ('decision making'), modulation, and background variables, such as hunger and satiety, can all be investigated in the same network [3,4]. In this review, we focus on the neural mechanisms for the rhythmic motor behavior, grazing, that underlies food ingestion in *Lymnaea *[5]. A regular program of rasps or bites allows the snail to ingest the uniform algal film upon which the snail often feeds. Food collected by rasping the edges or surfaces of floating plant material also is consumed by similar feeding movements [5]. The three movements that generate an ingestive feeding cycle (Figure 1A) occur as a continuous sequence, irrespective of the type of food substrate, and so constitute a single behavior. A central pattern generator (CPG) circuit generates the ingestive motor pattern [3]. The cellular organization of this CPG circuit and its control by various types of 'higher order' decision making and modulatory interneurons (Figure 1B) has been the major focus of research [3]. In other gastropods, such as *Aplysia*, ingestion of food pieces involves two behaviors, biting and swallowing and a third behavior, egestion (rejection of food), also is carried out by the same muscular organ [6]. The major aim in *Aplysia *has been to understand how different behaviors in the same general category can be generated by the same circuit. In this system, different motor programs are selected by combining activities in different types of descending higher-order interneurons called the cerebrobuccal interneurons (CBIs) [7]. Choice of behaviors by the CBIs occurs by the selection of other types interneurons, lower in a hierarchical architecture, that form a modular network. Each type of module implements a different type of motor pattern that underlie the three behaviors [8]. In another gastropod, *Pleurobranchaea*, the selection of alternative behaviors such as feeding and swimming (swimming inhibits feeding) has been the main interest and inhibitory synaptic interactions between different CPG circuits is the mechanism for behavioral choice [9].

There has been an evolution of ideas on the organization of the *Lymnaea *feeding system following the accumulation of more data on the sensory basis of feeding activation [4,10-14]. A previous hierarchical model [5] has been replaced by a distributed model in which individual neurons have shared and overlapping roles, with multifunctionality a common feature (summarized in Figure 1C). When sensory stimuli are applied to the lips and esophagus and compared with the results of 'artificial' neuronal stimulation by current injection, it was realized that direct feeding activation was widely distributed, not just to higher-order interneurons such as the CBIs, but also to CPG interneurons [15]. Thus the 'decision' to feed is not the property of a single class of hierarchically-organized neurons. In addition, rhythm generation is shared by CPG interneurons and motoneurons and modulatory functions are also widely distributed across the network [4,16,17]. A distributed type of organization also is found in other invertebrate motor circuits [18]. In the leech, the same group of interneurons fire during more than one behavior, indicating multifunctionality [19]. The decision to swim or crawl is carried out by a population of 'covarying' interneurons that show a pattern of activity that is specific to one of the two behaviors. Remarkably, manipulating the membrane potential of just one of these covarying neurons significantly biases the behavior towards either swimming or crawling depending on whether the cell is depolarized or hyperpolarized [20]. Within a CPG circuit, such as the pyloric CPG of the crustacean stomatogastric system, rhythm generation is not the property of any particular neuron but depends on a combination of endogenous plateauing and network synaptic connectivity that is distributed across the whole network [2,18].

## Background

**Figure 2:**
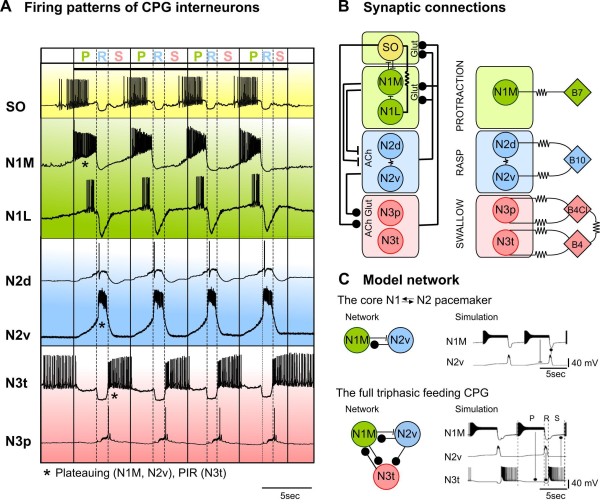
**Rhythm generation**. **(A) **Summary of firing patterns and endogenous properties interactions of the six different types of CPG interneurons in a SO-driven rhythm. The SO was depolarized for the duration of the traces (horizontal bar) to drive the feeding rhythm. Four cycles of feeding activity are shown with vertical solid lines dividing feeding cycles and vertical dashed lines separating out the protraction (P), rasp (R) and swallow (S) phases within each feeding cycle. The CPG interneurons fire during one of the three phases of the feeding cycle as indicated by the background colors. Asterisks indicate that the neuron has endogenous properties that contribute to network function **(B) **Synaptic connections and transmitters of the CPG interneurons and motoneurons. **(C) **Computer simulations of the two-cell (top) and three-cell (bottom) CPG networks. Dots, inhibitory synaptic connections; bars excitatory synaptic connections; resistor symbols, electrotonic synaptic connections. Abbreviations: Ach = acetylcholine; glu = L-glutamate; PIR = post-inhibitory rebound. See Abbreviations for all definitions of neuron types.

The analysis of feeding in *Lymnaea *began with electromygram (EMG) recordings and cinephotography of the buccal mass (feeding apparatus) [21]. This analysis revealed that ingestion is comprised of a repeated sequence of three distinct buccal mass movements, protraction, rasp and swallow, with a rest period between each cycle (Figure 1A). During each feeding cycle, the mouth opens and the toothed radula (or tongue) is scraped forward over the food substrate (the protraction phase of the feeding cycle). Food is then lifted into the mouth (rasp phase), which is closed while the food is being swallowed (swallow phase) and this sequence of movement is repeated during bouts of feeding that consists of up to 100 cycles [5]. Although the structure of the buccal mass is complex, consisting of 46 muscles, analysis is simplified by the finding that EMG and correlated motoneuronal activity is confined to one of the three phases of buccal mass movements shown in Figure 1A[21]. It was found that protraction, rasp and swallow phase muscles in the feeding apparatus are driven by a network of motoneurons (types B1 to B10; see Abbreviations section for all definitions of neuron type) [21-24] located in the buccal ganglia (Figure 1B) [21]. Four other types of rhythmically-active motoneurons (CV3, CV5 to CV7), located in the cerebral ganglia (Figure 1B), open and close the mouth [25]. Each phase of the feeding rhythm in all these motoneurons is generated by one of three main types of CPG interneurons N1 (protraction phase), N2 (rasp phase) and N3 (swallow phase) [22,26] providing sequences of excitatory and inhibitory synaptic inputs to motoneurons active in the different phases of the feeding rhythm (Figure 1C). The N1, N2 and N3 interneurons each have two subtypes N1M (medial), N1L (lateral), N2d (dorsal), N2v (ventral), N3p (phasic), N3t (tonic) (Figure 1B) and the firing patterns (Figure 2A), endogenous properties (Figure 2A), synaptic connectivity (Figure 2B, left) and transmitter content (Figure 2B, left) of these six types of neurons are known in considerable detail [26-30]. There are a variety of other neurons (Figure 1B), cerebrobuccal interneurons, slow oscillator (SO), octopamine-containing cells (OC) and cerebral giant cells (CGC) that control the output of the feeding CPG (Figure 1C) [3,31,32] and centrally-located esophageal mechanosensory neurons (OM) that respond to esophageal stretch [33]. In summary there are 9 types of CBIs (including the CGCs), 6 types of CPG interneurons, 15 types of motoneurons, 3 types of modulatory interneurons and 1 type of mechanosensory neuron making a total of approximately 100 neurons. There are likely to be yet more types of motoneurons as there are muscles deep in the buccal mass that have no known innervations [21] and mechanosensory neurons that respond to touch of the lips [34], movements of the buccal mass and radula that have not so far been investigated. Sensory inputs from peripheral chemoreceptors located in the lips and esophagus provide the stimulus for rhythmic feeding movements (Figure 1C) [35]. Touch provides a component of the food stimulus but rather being involved in the activation of feeding it strengthens the rasp phase of the feeding cycle when contact with the food substrate (Figure 1A, rasp) provides tactile input [34]. There are weak spontaneous feeding patterns that are observed both behaviorally and in isolated ganglia but these are slow and irregular compared with those observed in the presence of a strong feeding stimulus such as sucrose [3]. Continuous artificial stimulation of the SO is often used to drive a feeding rhythm (Figure 2A) in the isolated CNS [36]. Note that Figure 1C is an important reference for the rest of the review because it shows the network functions of the various types of neurons in the feeding circuit based on the current distributed model of the feeding network.

### Rhythm generation

Rhythmogenesis is not the property of a single class of neurons and CPG interneurons and motoneurons both contribute (Figure 1C). The major source of rhythmicity arises from the CPG interneurons. These interneurons fire in a three-phase sequence that is aligned to the feeding cycle (Figure 2A). The most important cells are the N1M and N2vs that occur as bilaterally symmetrical pairs. These cells form the core oscillator of the feeding CPG and alternate in activity during the protraction phase (N1M) and rasp phase (N2v) of the feeding cycle (Figure 2A). The rhythmic pattern of activity shown by the N1M and N2v cells depends on their plateauing properties and this provides the main oscillatory drive to the CPG network [28,30]. The recurrent inhibitory synaptic connections between the two cells (N1 → N2 excitation followed by delayed N2v → N1M inhibition) generate the sequence of N1M → N2v firing [37] (see model in Figure 2C, top). The N1Ms continue to show plateauing properties in cell culture [30] and so the plateauing is truly endogenous whereas the N2vs require the presence of a chemical modulator and are thus 'conditional' plateauing neurons [30]. In culture the N1Ms show long duration plateaus of up to 20 s in duration but in the intact network the inhibitory feedback from the N2vs reduces the duration to between 3 s and 10 s and causes an 'early' switch in the phases of the feeding pattern from protraction to rasp [30].

Evidence from resetting and photoinactivation experiments strongly support the hypothesis that the N1Ms and N2vs are the main generators of CPG rhythmic activity [37,38] but further validation of their role comes from recent computer modeling of the two-cell network [39]. Biophysically-accurate computer simulation of the N1M and N2v cells and 'connecting' them in a two-cell network generates a two-phase pattern of alternating rhythmic activity that mimics the main features of the biological system (Figure 2C, top) [39]. However, a three-cell network is required to get the triphasic feeding rhythm required for normal feeding behavior and this also was successfully modeled (Figure 2C, bottom) in the same study. This larger network includes the most important swallow phase interneurons, the paired N3ts. The N3t cell type is not an endogenous oscillator but fires by post-inhibitory rebound (post-inhibitory rebound (PIR), Figure 2A) [26] after receiving inhibitory synaptic input from the N2v interneurons (Figure 2C, bottom). By providing strong inhibitory feedback to the N1Ms during the swallow phase of the feeding rhythm, the N3ts delay the recovery of the N1Ms thus creating a separate swallow phase of the feeding cycle (Figure 2C, bottom). No inhibitory synaptic feedback is present to stop N2v firing and this is presumed to be due to an endogenous mechanism [37].

Other types of N cells, the N2ds and N3ps, are likely to play only a minor role in CPG oscillation because of their lack of endogenous properties [30]. They do, however contribute to network function because of the inhibitory synaptic feedback they provide to the N1Ms (Figure 2B, left). The N2ds appear to generate plateaus (Figure 2A) but these 'apparent' plateauing waveforms are due to the strong electrotonically-mediated synaptic inputs from the N2vs [28] rather than any endogenous plateauing capability [30]. The N3ps fire due to electrotonically-mediated excitatory inputs from the B4/B4Cl motoneurons that fire in the same swallow phase of the feeding cycle (Figure 2B, left) [30]. The N2ds do not show any endogenous oscillatory activity or PIR [30]. The N1L cells have more complex CPG-like and modulatory functions and their role will be considered later in the section on modulation.

More recently it was realized that some of the motoneurons play an important role in rhythm generation (Figure 1C) due to their electrotonic coupling with the CPG interneurons [24]. Previously, it was considered that motoneurons were follower cells of the CPG [3], with no influence on the generation of the feeding pattern. An important feature of the coupling is that it is restricted to motoneurons and CPG interneurons that fire in the same phase of the feeding pattern (Figure 2B, right). The B7 protraction phase motoneurons are coupled to the N1Ms, the B10 rasp phase motoneurons to the N2d/N2vs and the B4/B4Cl swallow phase neurons to the N3p/N3ts. This coupling contributes to same-phase synchronicity in the whole feeding network but also makes the motoneurons part of the CPG. This is because the motoneurons oscillate in a phase-locked manner with patterned output, provide functionally relevant synaptic inputs to the CPG interneurons and can reset the whole feeding pattern when they are manipulated within their physiological range [24]. The B7 motoneuron type is particularly important in rhythm generation. Making the B7 fire continuously by current injection activates a feeding pattern in inactive preparations by driving plateauing in the N1Ms. Conversely, long-duration suppression of spiking activity in the B7 by hyperpolarization completely stops a SO-activated feeding rhythm with loss of activity in the rest of the CPG, showing that the B7 is necessary for rhythm generation. Finally the endogenous properties of the motoneurons are also important in rhythmogenesis. Straub and Benjamin showed that the B4/B8 swallow phase motoneurons are capable of bursting in the absence of any synaptic inputs from the CPG interneurons and this provides an important mechanism contributing to rhythmicity as well. The bursting is induced by the release of 5-hydroxytryptamine (5-HT) from the CGC modulatory interneuron and so is conditional (see section on Modulation).

As in other systems [18], rhythm generation in *Lymnaea *depends a mixture of endogenous (bursting, plateauing and PIR) and network synaptic properties (recurrent inhibition, electrotonic coupling) both of which are widely distributed across the CPG/motoneuronal network. The data indicate that the feeding motoneurons in *Lymnaea *have dual roles in rhythm generation and control of movement (Figure 1C) and so they cannot simply be followers of the CPG interneurons, as suggested by the previous hierarchical model [5]. Motoneurons are also notable for their contribution to rhythm generation in other motor networks in both invertebrates and vertebrates (reviewed in [24]) exemplified by the stomatogastric system of crustaceans where the motoneurons are the CPG [2].

### Initiation

**Figure 3:**
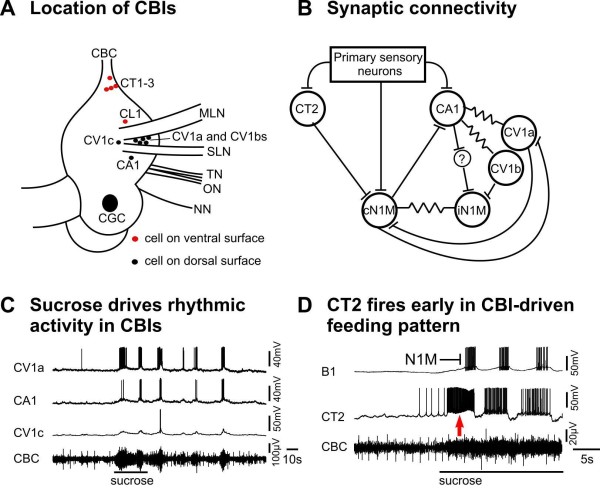
**Locations, synaptic connectivity and sucrose responses of the cerebrobuccal interneurons (CBIs)**. **(A) **Cell body locations of the 13 CBIs in the right cerebral ganglion. A similar population of cells occurs on the left side **(B) **Electrotonic synaptic connections (resistor symbols) between the CBIs (CA1, CV1a, CB1b types) and excitatory chemical synaptic connections (bars) between the CBIs and the N1M CPG interneurons, contralateral (cN1M) and ipsilateral (iN1M). Note that the CBIs can drive the N1Ms (see text) but they also receive excitatory feedback from the N1Ms. **(C) **Rhythmic responses to sucrose application to the lips recorded intracellularly in three individual CBIs but also extracellularly from their axons in the cerebrobuccal connective (CBC). Suppression of activity in these CBI by intracellular hyperpolarization (not shown here) shows that there were further types of CBIs contributing to the population response recorded in the CBC. **(D) **The CT2 is the first to fire in a sucrose-driven rhythm (arrowed) and its strong burst of activity precedes the first burst of spikes in the B1 feeding motoneuron. The B1 burst is known to be driven by monosynaptic excitatory synaptic inputs from N1M CPG (bar). This indicates that CT2 fires before the N1M. See Abbreviations for all definitions of neuron types.

Higher order neurons that can drive CPG activity and respond to sensory cues necessary for the behavior are considered to be good candidates for initiation of rhythmic motor behaviors [40]. A number of CBI neurons with these characteristics have been identified in the cerebral ganglion of the *Lymnaea *feeding system (Figure 3A) and they have a major role in CPG initiation. The axons of the CBIs project from the cerebral to the buccal ganglion where they have synaptic connections with the CPG (Figure 3B). The previously described CV1a and CV1b cells [41] and the newly discovered CA1 and CT2 cells [32] are the most interesting CBI cells in terms of the initiation of feeding activity. Since these neurons exist as bilaterally symmetrical pairs and there are up to three CV1bs on each side (Figure 3A), this represents a population of at least ten cells that have the potential to be involved in chemosensory-induced feeding. Sucrose application to the lips induces simultaneous spiking activity in all these cells (examples in Figure 3C). This is recorded at the population level by extracellular recordings on the CBC [35] and confirmed by intracellular recordings from individual cells (Figure 3C). Blocking polysynaptic pathways has no effects on CBI activation by sucrose [32] so it appears that the primary chemosensory pathways originating in the lips have direct monosynaptic connections with the CBIs (confirmed by anatomical data in [32]). The simultaneous activation of the CBIs by food and the demonstration that each of the cells can individually activate feeding (see below) suggest that the CBIs contribute to feeding activation as a group.

Early experiments [41] showed that electrical stimulation of the CBI type, CV1a, could initiate and maintain a robust feeding rhythm due to its strong monosynaptic excitatory connection with the N1M CPG interneurons. Once the feeding rhythm has started, the CV1a cells receive inhibitory synaptic feedback from the CPG that makes them fire phasically in the same protraction phase of the feeding cycle as the N1Ms [41]. The CV1b cell type has a weaker effect on the feeding CPG and its firing pattern tends to be more or less continuous and less modulated by feedback from the CPG compared with the CV1as [42]. The role of the more recently discovered CA1 cells is likely to be linked to the CV1 network [32]. The cell is electrotonically coupled to both the CV1a and CV1b cell types and the cells fire together in the same phase of the feeding network (Figure 3B). Like the CV1a, the CA1 can initiate and maintain a feeding rhythm when electrically stimulated by current injection although unlike CV1a its connection with the N1M is polysynaptic (Figure 3B). When the cells were recorded together, a variable order of activation was observed suggesting that neither cell is predominant in activating feeding [32]. A further type of CV cell type, CV1c has been found (Figure 3Aand [32]) that is also electrotonically coupled to the CA1 and CV1a cells. It tends to fire weakly in a sugar-driven feeding rhythm (Figure 3C), perhaps due to its coupling with these other cells. Other CBIs (CL1, CT1 and CT3) also respond to sugar stimulation and contribute to the CBC-recorded population response [32] but as they have no clear role in activating feeding they have not yet been investigated in detail. Further types of CBI function such as behavioral switching [43] and feed-forward control of phase duration [44] have been demonstrated by elegant costimulation experiments in the related mollusk *Aplysia *and some of these functions also may be present in *Lymnaea *[10].

The most recent type of CBI to be investigated, the CT2, is considered to be the most important in starting the pattern of feeding activity in the CPG [32]. This cell shows a strong burst of spikes within 1 to 2 s of application of sucrose to the lips (Figure 3D, arrowed) unlike the CV1a and other CBIs that slowly depolarize over several seconds before firing. The protraction phase CPG interneuron N1M previously was shown to be the first cell to fire in a sucrose-driven pattern [10] ahead of the CV1a. However, extracellular recordings of the CBC show that the CT2 is active before the N1M [32]. The recording in Figure 3D (horizontal line and bar) shows that the first burst of spikes in the B1 motoneuron, driven by N1M synaptic inputs [45], occurs after the first burst of spikes in the CT2 confirming the CT2, N1M order of firing. The CT2 can drive activity in the N1M [32] so the early sucrose-driven burst in the CT2 is likely to be critical in triggering the N1M to fire at the onset of a sequence of feeding cycles. The other CBIs fire slightly later usually during the second cycle of feeding activity and then contribute to N1M plateauing [10].

The N1Ms also play an important role in feeding activation (Figure 1C). There are two chemosensory pathways for sucrose activation of the feeding CPG that both converge on the N1Ms: the first provides early excitation of the CT2s which then stimulate activity in the N1Ms (above), the second provides direct monosynaptic excitatory input to the N1Ms as shown by its persistence during the blocking of polysynaptic pathways [25]. Thus, rather than one of these pathways dominating, there is a coactivation of the CBIs (not just the CT2s, Figure 3C) and the N1Ms by primary chemosensory neurons. The stimuli from both routes are then integrated by the N1Ms to give rise to rhythmic feeding by triggering N1M plateauing. As the N1Ms are rarely spontaneously active [30] the triggering by excitatory synaptic inputs, direct and indirect, is essential for feeding to occur. In our computer simulation of the feeding network (Figure 2C) the N1M was 'artificially' depolarized to mimic the triggering effects of these two types of excitatory inputs [39].

**Figure 4:**
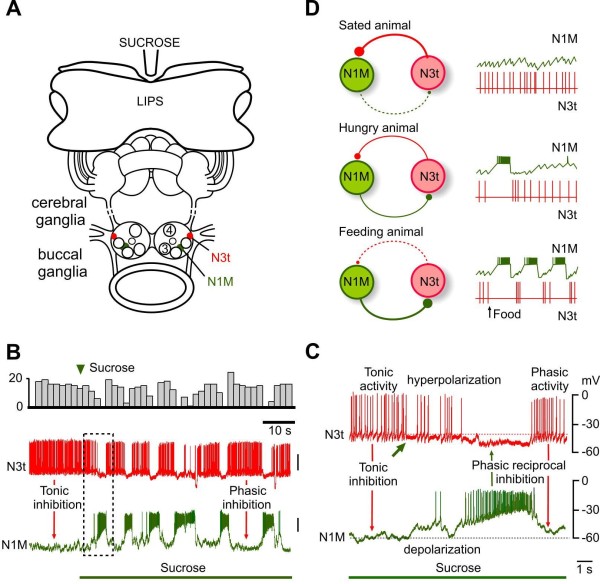
**N3t interneuron has multiple roles in the feeding system**. **(A) **The semi-intact preparation used for electrophysiological recording and sucrose stimulation showing the location of the feeding interneurons, N1M and N3t. **(B) **An experiment showing that the food stimulus, sucrose, reduces the suppressive inhibitory control of the N3t cell and releases rhythmic fictive feeding. It is therefore important in switching the feeding system from quiescence to feeding. When it changes from tonic firing to rhythmic activity it becomes part of the CPG. The change in the pattern of activity is emphasized by the top trace where the number of spikes is accumulated in 3 s bins. **(C) **Expansion of the boxed area in (B) shows the first cycle of fictive feeding activity in the N1M after the sucrose-induced reduction in N3t firing rate. The arrow under the N3t trace indicates the point at which N3t starts to hyperpolarize and its tonic firing begins to decrease. This decrease in firing is followed by complete suppression of N3t firing when the N1M becomes active and synaptically inhibits the N3t. The subsequent phasic N1M-N3t reciprocal inhibition leads to alternating patterns of N1M/N3t firing seen throughout the feeding pattern that follows. **(D) **Hunger and satiety influences spontaneous feeding activity by influencing the level of tonic inhibition of the feeding CPG interneuron N1M. In satiated animals, the N3t fires continuously and the consequent inhibitory effects on the N1M prevent spontaneous feeding. In hungry animals, even with no food present, there are occasional feeding bursts in the N1M due to the lower rate of firing in the N3t. In feeding animals the tonic N3t firing is weak and insufficient to prevent sustained bursting in the N1M. Dots indicate inhibitory chemical synaptic connections. Adapted from [26] with permission from Elsevier. See Abbreviations for all definitions of neuron types.

The above account reveals that feeding initiation depends on integrating the excitatory synaptic effects of convergent chemosensory pathways. However, there is also an opposing inhibitory mechanism intrinsic to the CPG that suppresses feeding. This inhibition has to be overcome before feeding can occur. In the absence of food, particularly in satiated animals (see the Hunger and satiety section, below), snails show long periods of quiescence with only occasional spontaneous rasps. It has been shown that the quiescence is due to tonic inhibition of the N1Ms by the N3ts [4]. During quiescence the N3ts fire continuously and via the strong inhibitory connection prevent N1M plateauing (Figure 4B, left). When sucrose is applied to the lips (Figure 4A), the N3ts are hyperpolarized (Figure 4C) reducing the level of tonic inhibition to the N1M and this has a permissive effect in allowing the N1M to plateau (Figure 4C). Thus during the sucrose-driven feeding pattern, the N3ts fire rhythmically as part of the feeding CPG (Figure 4B, right) due to the reciprocal inhibitory synaptic connections with the N1Ms. Thus N3ts have a role in modulating the feeding network as well as being part of the CPG (Figure 1C).

The suppression of motor circuits by tonic inhibition has been observed in other episodic motor systems [46]. An obvious general function is to prevent unnecessary non-goal-directed activity that would be energetically expensive. The reason why rhythmic motor programs might be a particular target for tonic inhibition is that many of them involve CPGs that are often 'spontaneously' active and therefore need to be 'actively' suppressed for energy saving.

### Modulation

As part of the consideration of the dynamic control of the feeding network, the role of several types of modulatory neurons will be considered next. One type of modulatory interneuron (the CGC) is clearly 'extrinsic' to the CPG network and regulates its responsiveness, a type of 'gating' function. Other types of modulatory neurons (SO, OC and N1L) are considered to be 'intrinsic' to the CPG. This type of modulation is inferred from the close relationship the modulatory neurons have with the CPG [18]. In *Lymnaea*, intrinsic modulation is important in reinforcing, maintaining and controlling the frequency of the feeding rhythm.

#### Cerebral Giant Cells

**Figure 5:**
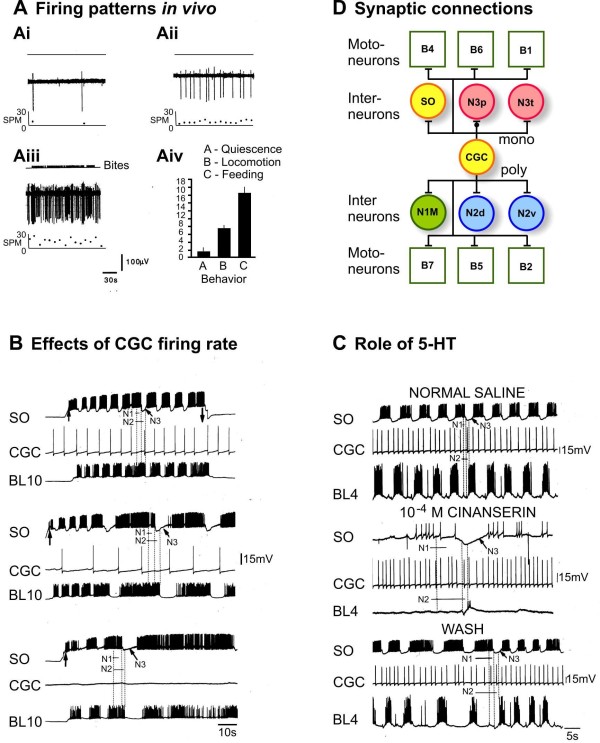
**Extrinsic modulation of the feeding network by the cerebral giant cells (CGCs)**. **(A) **Firing rates of a CGC recorded in a freely moving intact animal using fine wires attached to the cell body. The bottom traces show the instantaneous firing rate in spikes per minute (SPM). The CGCs fired rarely during quiescence (approximately 1 SPM, Ai), at higher rates during locomotion (approximately 7 SPM, Aii) but only when the CGCs fire at approximately 15 SPM (Aii) did the animal show any feeding behavior. These differences in firing rate were consistent in the eight animals that were recorded (mean rates plotted in Aiv). **(B) **Electrophysiological correlates of varying CGC firing rates. The firing of the CGCs were artificially set at firing rates that corresponded to those recorded in the intact animal and the feeding responses to SO stimulation tested by recording fictive feeding in a B10 motoneuron. At the highest rate of CGC firing (15 SPM, top trace), the SO could drive a fast rhythm that was equivalent to that recorded in food driven rhythms in the intact animal. With lower rates of CGC firing (7 SPM) the rhythm was much slower (middle trace) and in the absence of CGC firing very little fictive feeding activity could be observed apart from a few slow cycles at the beginning of SO stimulation (bottom trace). **(C) **The serotonin receptor antagonist, cinanserin, reversibly blocks the modulatory effects of CGC firing in a SO-driven feeding rhythm. The CGCs are the only neurons in the feeding network that are serotonergic. **(D) **The CGCs have monosynaptic (mono) and polysynaptic (poly) connections with CPG interneurons and motoneurons of the feeding network. These are excitatatory (bars) apart from the N3ps that have a dual inhibitory/excitatory (dot/bar) synaptic response to CGC stimulation. See Abbreviations for all definitions of neuron types.

The paired serotonergic CGCs (location in Figure 1B) play a gating or permissive role in the generation of feeding behavior but cannot drive activity in the CPG at physiological rates of firing [16]. Continuous or tonic spiking activity in the CGCs provides a background of excitatory modulation to the feeding network that lowers the threshold for activation to 'permit' a feeding response. By recording the CGCs in the intact animal using fine wire recording [16], it was found that during feeding the CGCs fire maximally in the 7 to 20 spike/minute range (Figure 5Aiii) and below this range of firing, for instance during locomotion (Figure 5Aii) and quiescence (Figure 5Ai), feeding does not occur. Above the threshold level of firing, the CGCs also influence the frequency of the feeding rhythm, a second type of modulation [16,17]. The importance of CGC firing rates in maintaining and controlling the frequency of the feeding rhythm was confirmed electrophysiologically in a SO-driven feeding rhythm (Figure 5B). 5-HT (the CGC's transmitter) is required for CGC modulation [16] and this is shown in pharmacological experiments where the 5-HT_2 _receptor antagonist, cinanserin, reversibly blocked a SO-driven rhythm (Figure 5C).

The role of the CGCs in gating depends on two types of mechanisms. One involves background synaptic depolarization of CPG interneurons and motoneurons by tonic firing in the CPGs (Figure 5D) [30]. The other depends on the effects of CGC firing on the endogenous properties of CPG interneurons and motoneurons [17,30]. The most significant CGC synaptic connections are with the N1M and N2v cells [17]. The CGCs reduce the threshold for plateauing in both of these neuron types [17] and this plays a critical role in network gating because of the major role these cells play in rhythm generation (above). The N1Ms are slowly depolarized by CGC stimulation and this makes them more likely to respond to other types of triggering synaptic input from cells such as the CBIs. The N2vs are also depolarized by the CGCs. This is usually too weak to initiate plateaus but if the N2vs are depolarized by spontaneous synaptic input or by artificial depolarization then plateaus are initiated as is the case with the N1Ms. However, the longer-term effects of CGC tonic firing may be most significant for the N2vs role in gating [17]. This was clear in suppression experiments where after 2 minutes of spiking loss in the CGCs the N2vs cease to plateau, recovering when CGC firing is allowed to recommence. This experiment indicates that spiking activity in the CGCs is required for the endogenous plateauing of the N2vs.

The post-synaptic effects of CGC tonic firing on motoneurons are also important in network gating [47]. The resulting tonic depolarization of motoneurons reduces their threshold for spiking and makes them more responsive to the CPG synaptic inputs that drive their rhythmic activity [3]. Without this modulatory synaptic input the motoneurons do not fire enough to drive muscular activity. However, because of the electrotonic coupling between the motoneurons and CPG interneurons (Figure 2B, right), CGC-induced increases in motoneuronal spiking activity also contribute to gating at the CPG level [48]. Gating of network activity also is due to CGC modulatory effects on the endogenous properties of motoneurons. In culture and in the intact ganglion, the firing of the CGCs or application of their transmitter 5-HT for a few seconds cause multiple modulatory effects on rasp and swallow phase motoneurons that last for many minutes. The resulting membrane potential depolarization, induction of endogenous bursting and an enhancement of PIR [48] all increase the probability of motoneuron firing during feeding cycles contributing significantly to the gating function of the CGCs at both the motoneuron and CPG levels [48].

Frequency control depends on the CGC excitatory synaptic connections with the N1M CPG interneurons (Figure 5D) and on the endogenous modulation of motoneuron bursting by the CGCs (above). Increasing the CGC firing rate in the 1 to 40 SPM firing range results in a linear increase in the frequency of the feeding rhythm and this is due to a reduction in the duration of the N1M/protraction phase of the feeding cycle [17]. At higher rates of CGC firing, the amplitude of the post-synaptic depolarization in the N1M is enhanced causing the earlier triggering of plateaus and a consequent reduction in the duration of the protraction phase of the feeding cycle [17]. Stimulating the CGCs also increases the frequency of bursting in swallow phase motoneurons [48] and so frequency control happens at the motoneuronal level as well.

#### Slow Oscillator

In a number of invertebrate systems, modulatory interneurons that drive CPG activity receive feedback from the CPG they control resulting in their spike activity being entrained to the motor rhythm. It has been suggested that this type of reciprocal interaction provides positive feedback within the network that has a role in maintaining rhythmic patterns [49]. Evidence for this type of mechanism exists in the *Lymnaea *feeding system. Neurons such as the SO (a single cell, Figure 1B) can initiate feeding patterns in the CPG when artificially stimulated into tonic activity by current injection [50]. Once the feeding rhythm commences in the CPG, the SO becomes rhythmically active (Figure 2A) due to synaptic inhibitory feedback from CPG interneurons such as the N2vs (Figure 2B, left). The SO has no endogenous capability to oscillate [30]. The SO has strong excitatory monosynaptic connections with the N1M CPG interneuron [36,50] and fires just before it in the same protraction phase of the feeding cycle (Figure 2A). The SO thus provides a component of the depolarizing synaptic input that triggers the N1M plateau. Suppressing activity in the SO by current injection in a sucrose-driven rhythm does not prevent the occurrence of a feeding pattern in the CPG [50], indicating that it is not part of the CPG. However, in the absence of SO spiking, rhythmic activity in the CPG is not maintained. It slows in frequency and becomes irregular [10]. It has previously been shown [50] that the SO controls the frequency of CPG oscillation when stimulated to fire at different rates by current injection so both frequency control and maintenance of the regular feeding pattern depend on the SO. This data indicate that the core N1M-N2v oscillator alone is not able to generate the regular, high frequency pattern observed with strong feeding stimulus such as sucrose in the intact animal. This was not unexpected since previous attempts to drive the CPG by 'maximum' current injection into the N1M showed that it is incapable of generating high frequency feeding patterns, unlike the SO [36]. This ability of the SO to maintain and control the frequency of the CPG rhythm by changing the duration of the protraction phase of the feeding cycle was reproduced by computer modeling of the synaptic connections and firing patterns of the SO and CPG interneurons [39] that had been revealed by electrophysiological recording.

These results on the SO are incompatible with the hierarchical model of the feeding network which had suggested that the SO was a command-like neuron involved in feeding initiation [5] but rather indicate that it is part of the modulatory network controlling feeding (Figure 1C).

#### N1L

**Figure 6:**
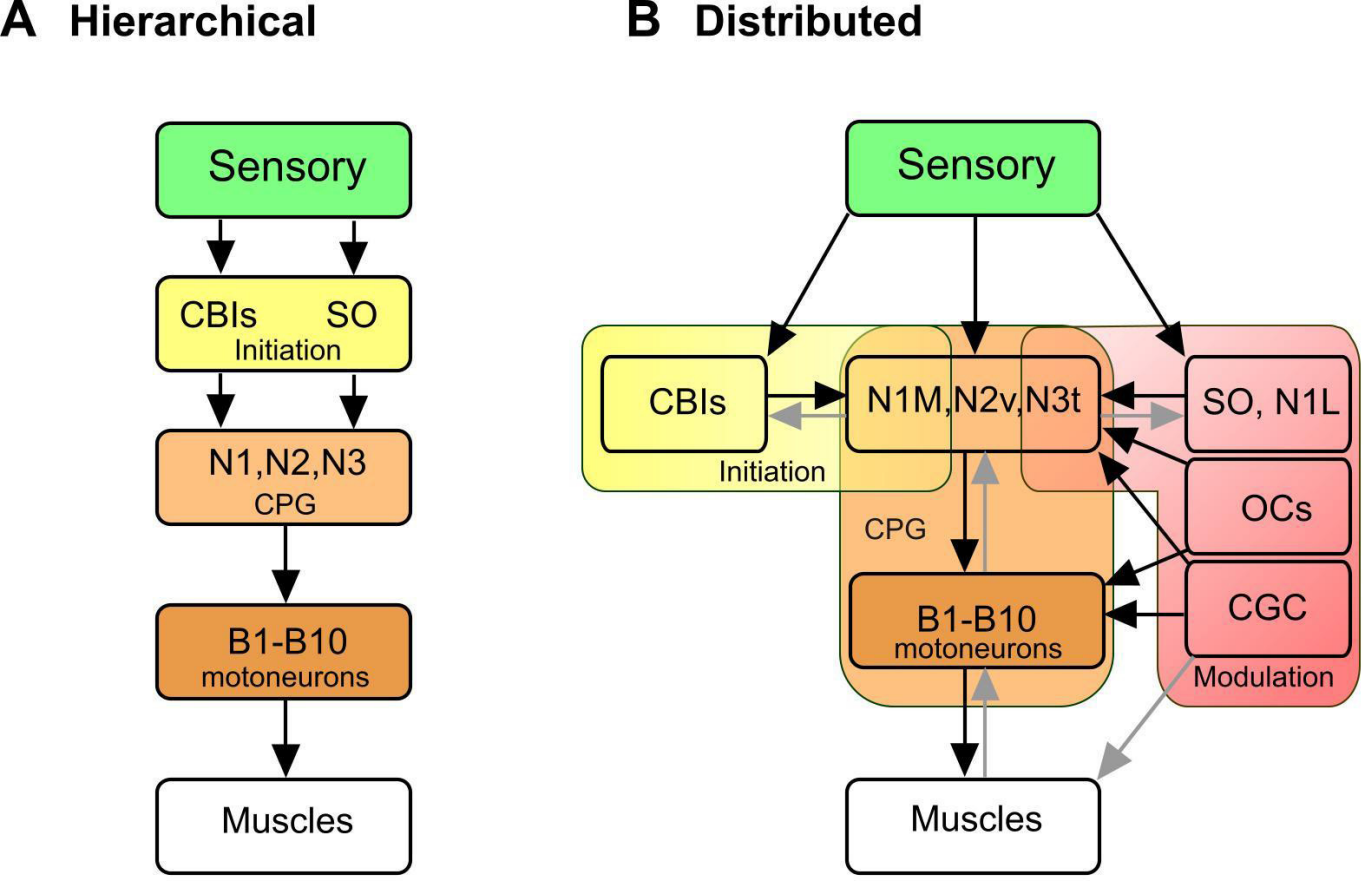
**Alternative schemes for the organization of the *Lymnaea *feeding system**. **(A) **In a previous hierarchical model, the command neurons CBIs and the SO were believed to be responsible for activation of the CPG (N1, N2, N3) following sensory stimulation. The CPG then drives rhythmic activity in the motoneurons (B1 to B10) to elicit muscular contractions and the movement pattern. (B) The current model suggest that the sensory activation of the system is organized in a more distributed manner, and the CBIs and SO, although possessing potential to act as command-like neurons are part of a more complex system for the initiation and modulation of the feeding network. The CBIs act together with the N1Ms to initiate feeding (yellow box). The SO is thought to be mainly involved in maintaining a strong feeding rhythm rather than initiation and is therefore modulatory (pink box). Other modulatory neurons, for example, the CGCs and the OCs, control other aspects of feeding output (pink box). As indicated by the high degree of reciprocal synaptic connectivity between the controlling elements, no one cell type can be considered to uniquely activate the feeding CPG and none of them act at a particular 'level' to indicate a hierarchical type of organization. A similar lack of hierarchical organization is also indicated for the CPG (light orange box). The CPG consists not only of N1M, N2v and N3t interneurons but also the motoneurons (B1 to B10). The motoneurons are not 'followers' of the N cells at the bottom of a hierarchy but act as part of a distributed CPG network. Light arrows indicate feedback connections in the network or an unproven connection in the example of the CGC. See Abbreviations for all definitions of neuron types.

The paired N1Ls fall outside the simplest classification of intrinsic modulation having some properties that are CPG-like and others that are modulatory [51]. The N1L is part of a group of neurons that fire during the protraction phase of the feeding rhythm (Figure 2A) and its function is best understood by comparing its properties with the N1M and the SO that fire during the same phase. Unlike the N1M, the N1L does not have any plateauing or other endogenous properties to suggest that it is part of the core oscillatory mechanism. However, its firing is necessary for rhythm generation and in this respect it is similar to the N1M. Thus when N1L spiking is artificially suppressed in a sucrose-driven feeding pattern, activity in the CPG network, including the N1Ms, ceases [51]. This is not the case with the SO, where suppression of activity only leads to a slowing of the rhythm (see above). It appears that the main function of the N1L is to reinforce activity in the protraction phase of the feeding cycle due to the strong excitatory monosynaptic connection with the N1Ms. Irrespective of the size of N1L current injection, this connection drives a high-frequency (3 to 5 s period) feeding pattern in the N1Ms. The strong electrotonic synaptic connection between the SO and N1L suggest that the cells normally act together: the N1L to produce a strong activation of the protraction phase of the feeding rhythm, the SO to maintain and control the frequency of the feeding rhythm. The SO frequency control function depends on the progressive facilitatory effects that a train of SO spikes has on N1M excitatory postsynaptic potential (EPSP) amplitude [36,50]. Temporal summation of these facilitatory EPSPs is greater when the SO fires at a higher rate allowing the N1M to reach the threshold for plateau formation earlier in the feeding cycle. This reduces the duration of the protraction phase with a consequent increase in cycle frequency. In contrast, the N1L to N1M EPSPs do not facilitate. The high level of synaptic connectivity with other CPG interneurons and its requirement for CPG rhythm generation suggest that the N1L is part of the CPG. However, the ability to control a particular phase of the feeding rhythm and to drive a feeding pattern (like the SO) suggests a more modulatory role. Also the N1Ls have few synaptic connections with motoneurons, unlike the N1Ms and N2vs CPG interneurons, again resembling the SO. This data suggest a hybrid modulatory/CPG function for the N1Ls. This is interesting because it may be indicative of an evolutionary process that allowed stereotyped motor patterns to become more flexible [51]. This also could be true for other CPG systems where intrinsic neurons have joint roles [52]. To reflect this role in the controlling the flexibility of the CPG circuit the N1L has been included in the modulatory rather than CPG category in the summary of Figure 6B.

#### Octopamine-containing cells

The three OCs are not considered to be part of the CPG because a basic feeding rhythm can occur when they are silent. However, they definitely modulate the feeding network. They receive rhythmic synaptic inputs from the CPG during fictive feeding in isolated preparations and have synaptic output connections with the CPG interneurons (Figure 1C) although not with the CGCs [53]. The function of the OCs is to augment the ability of the feeding CPG and CBIs to generate strong high frequency rhythms. The most interesting feature of OC function is the ability to provide long-duration 'polycyclic' modulation of the feeding CPG. Single or multiple 'prepulse' stimulation of an OC cell or application of octopamine facilitates the subsequent response to SO [54] or food stimulation [53] over many cycles. This contrasts with the SO whose modulatory effects are 'intracyclic' acting repeatedly during a sequence of feeding but having a duration that is always within one cycle. This long duration feeding response from OC stimulation is mainly due to slow depolarizing synaptic responses that the OCs have on protraction-phase neurons such as the N1Ms, N1Ls and the SO whose effects extend over several feeding cycles. With repeated stimulation of the OCs, these depolarizing synaptic responses are facilitated and can drive the N1Ms into prolonged rhythmic plateauing. Another component of the polycyclic mechanism derives from the ability of the OCs to increase the strength of the synaptic excitatory output connections that the SO and N1L have with the N1Ms [55]. Like the direct effects of the OCs on the N1M, these heterosynaptic effects of OC stimulation are facilitated by repeated stimulation of the OCs. The suggested mechanism is presynaptic facilitation as the SO neurons have lower action potential threshold after the end of OC activity [55]. As well as influencing the strength of synaptic connections OCs/octopamine also increase the endogenous excitability of feeding motoneurons and interneurons making them more responsive to synaptic input. In two cell types, the B1 and B4 motoneurons [56], octopamine increases the size of a fast inward sodium current and this accounts for the ability of the cells to generate more action potentials to a standard depolarizing stimulus.

### Hunger and satiety

Another example of modulation that contributes to the flexibility of feeding behavior is hunger and satiety, a motivational/behavioral state mechanism. One type of satiety mechanism controls the frequency of spontaneous feeding cycles and a second type controls meal length.

Hunger and satiety have no effect on the feeding responses to a strong feeding stimulus such as sucrose but they do have an influence on the frequency of spontaneous rasping movements that occur in the absence of food. These are greater in starved compared with well-fed snails [57]. Spontaneous feeding movements are considered to be part of appetitive exploratory behavior and would be expected to increase in hungry animals. By recording the patterns of 'fictive feeding' activity from motoneurons in isolated brains it was possible to find a neural correlate of this motivational effect, so that preparations made from hungry snails were more likely to show high frequency bouts of fictive feeding activity [57]. Given that the neural correlate of hunger and satiety was recorded in a completely isolated ganglion, the effects must be due to a central mechanism that controls spontaneous feeding activity. One of the central controllers of spontaneous feeding is the N3t CPG interneuron and this cell is involved in mediating the effects of hunger and satiety. As was described earlier, the N3ts fire tonically to inhibit the N1M cells and the rate of this tonic activity determines the level of activity in the whole feeding CPG. By comparing the rates of firing in isolated ganglia it was found that the N3t firing frequency was higher in satiated compared with starved snails and that this was inversely correlated to the frequency of spontaneously fictive feeding cycles [4]. Thus one of the mechanisms for control of spontaneous feeding by hunger and satiety is due to the regulation of tonic inhibitory inputs to the CPG (Figure 4D).

However, the role of inhibition in hunger and satiety control is not confined to the CPG. There are large spontaneous inhibitory postsynaptic potential (IPSP) inputs recorded in the CV1a (CBI) interneurons that control their level of activity. Like the N3ts, the frequency of this inhibitory input is significantly greater in satiated compared with hungry animals [58]. This inhibition of the CBIs recently has been found to originate from the peptidergic pleural interneuron (PlB, pleural-buccal neuron) that has widespread inhibitory effects on the feeding circuit [59]. Like the N3t the PlB cell fires in a tonic pattern and presumably its spiking activity is modulated by hunger and satiety, although this remains to be confirmed by direct recording.

Another type of satiety mechanism relies on sensory activation of mechanoreceptors that respond to the distension of the esophagus [33]. From three to five esophageal mechanoreceptors (OMs) occur in each buccal ganglia (one is shown in Figure 1B to indicate location) and their axons project to the proesophageal region of the gut that lies immediately behind the buccal mass. The EMs respond to experimental gut extensions with high frequency firing suggesting that they are involved in detecting the presence of food in the gut that is normally distended by the ingestion of bulk plant material. The EMs adapt their firing rates during artificially maintained distensions but the rhythmic gut movements observed during peristaltic movement of food through the gut are likely to maintain activity in the EMs. The EMs have extensive synaptic connections with neurons of the feeding circuit and they inhibit several types of CPG interneurons and the SO [33]. Stimulation of a single EM is sufficient to slow or inhibit a SO-driven rhythm providing the appropriate inhibitory effects on the feeding circuit to mediate satiety. Therefore it seems likely that the EMs act to control the level of food intake to control meal or bout length. They also could prevent overloading of the gut. The satiety mechanism based on EM mechanical stimulation relies on the continued presence of sensory input and appears to be distinct from the N3t-mediated satiety mechanism that persists in the isolated ganglia.

## Conclusions

The evidence reviewed here supports a distributed type of network organization (Figure 6B) rather than a linear hierarchical model (Figure 6A).

### Initiation of feeding

In the hierarchical model, the SO and CBI interneurons (for example, CV1a) were considered to be at the top of the hierarchy acting as command cells for the initiation of feeding (Figure 6A). This model originated from experiments where activation of the SO and CV1a by intracellular current injection in isolated CNS preparations was shown to initiate and maintained a fictive feeding rhythm [41,50]. The detailed phase characteristics of these SO and CV1a-driven rhythms resembled those recorded in the semi-intact preparation when feeding movements of the buccal mass were initiated by sugar applied to the lips. CV1a and SO alone could both drive a feeding rhythm because of their monosynaptic excitatory connections to the N1M neurons of the CPG. They did so independently because there were no synaptic connections between the two cell types [41]. Subsequent suppression experiments when chemical stimuli were used to drive feeding patterns showed that the SO was not necessary for feeding initiation but had a modulating role in maintaining and controlling the frequency of the feeding pattern [10]. The CV1a and other CBIs were subsequently confirmed to be part of the initiation system by showing that are consistently active during food-activated rhythms and that their activity is necessary for the early activation of feeding patterns in sucrose-driven rhythms [10] (Figure 3). However, the CPG interneurons like the N1Ms also receive direct chemosensory input from the lips and this can activate a feeding rhythm independently of the CBIs [15] so the CBIs do not act alone in feeding activation. This data indicate that feeding initiation is distributed between the CBIs and N1Ms (Figure 6B) and that their relationship is non-hierarchical. In this new model, the SO cell is considered to be part of a distributed modulatory system (Figure 6B).

### Rhythm generation

The role of the CPG interneurons in rhythm generation is clearly established. The generation of the rhythm depends on the plateauing properties of the N1Ms and N2vs and post-inhibitory rebound in the N3ts [26,30]. The sequence of firing in these cells is determined by their synaptic connectivity so the CPG firing pattern depends on both endogenous and network properties distributed across the whole CPG interneuronal network. More detailed studies on the role of the motoneurons made it clear that they are part of the rhythm generation mechanism as well as generating muscle contractions [24]. They are therefore not just followers of the CPG interneurons as indicated in the hierarchical model (Figure 6A) but are multifunctional and form part of a distributed CPG network as well (Figure 6B).

### Modulation

Specifying the network roles of modulatory neurons is more difficult because of their diverse and overlapping functions. However it is clear that the distributed model of the feeding network fits them well (Figure 6B). The CGCs are extrinsic to the core CPG circuit and are involved in both gating and frequency control, sharing the latter role with the SO. The N1L cell type appears to have several functions in the feeding network [51]. It has intimate synaptic connections with the rest of the CPG network indicating that it might be part of the CPG but unlike a CPG interneuron like the N1M it can drive a strong feeding activity in the feeding network [51]. It is suggested that this is due to modulatory effects on the protraction phase of the feeding rhythm produced by a strong chemically-mediated synaptic connection with the N1M interneurons. The OC interneurons also have complex connections with the rest of the feeding network [31], without appearing to be part of the CPG. Like the SO they appear to be involved in maintaining and strengthening rhythmic activity, but unlike the SO this involves polycyclic synaptic and modulatory effects on the majority of cells in the feeding network giving the cells a global role in network function [31]. The N3ts are particularly interesting in that they are a target for central behavioral state control mechanisms (Figure 4), as well as having a dual role in behavioral switching and pattern generation [4]. The periods of quiescence shown by the *Lymnaea *between bouts of feeding movements are due to tonic inhibition of the feeding CPG by the N3ts that is dominant in the absence of a food stimulus or when the animal is satiated (Figure 4). N3ts thus have state-dependent modulatory inhibitory functions controlling activity in the CPG as well being a member of the CPG (Figure 6B) and are the epitome of a multifunctional neuron.

### What are the advantages of having a distributed network?

Recording experiments show that neurons vary a lot in their detailed firing patterns for instance the number of spikes in a burst and the strength of synaptic connections vary a lot from preparation to preparation [5,23]. Having complimentary and potentially alternative neuronal substrates for network functions may be a mechanism for improving the robustness of behavioral responses in what is a 'noisy' network. In a pure example of a distributed network, all the neurons of the network would contribute to all the required network functions but this is not the case in *Lymnaea *where a more limited or partial type of distributed organization exists with some specialization of function. It can be argued that this type of distributed network that has evolved for *Lymnaea *feeding offers advantages from the point of view of robustness without the need for excessive redundancy that occurs in wholly distributed networks where there are often duplicate sets of similar neurons. Sharing functions in a distributed network also would be 'economically' sensible in the *Lymnaea *feeding system where only approximately 100 neurons are available to carry a variety of complex tasks such as decision making, rhythm generation, motivation and memory formation [11-13].

## Abbreviations

B1: Buccal 1 (motoneuron); B2: Buccal 2 (motoneuron); B3: Buccal 3 (motoneuron); B4: Buccal 4 (motoneuron); B4Cl: Buccal 4 cluster (motoneurons); B5: Buccal 5 (motoneuron); B6: Buccal 6 (motoneuron); B7: Buccal 7 (motoneuron); B8: Buccal 8 (motoneuron); B9: Buccal 9 (motoneuron); B10: Buccal 10 (motoneuron); CA1: Cerebral anterior 1 (cerebrobuccal interneuron); CBIs: Cerebrobuccal interneurons; CGC: Cerebral giant cell (modulatory interneuron); CL1: Cerebral lateral 1 (cerebrobuccal interneuron); CT1: Cerebral tentacle 1 (cerebrobuccal interneuron); CT2: Cerebral tentacle 2 (cerebrobuccal interneuron); CT3: Cerebral tentacle 3 (cerebrobuccal interneuron); CV1a: Cerebral ventral 1a (cerebrobuccal interneuron); CV1b: Cerebral ventral 1b (cerebrobuccal interneuron); CV1c: Cerebral ventral 1c (cerebrobuccal interneuron); CV3: Cerebral ventral 3 (motoneuron); CV5: Cerebral ventral 5 (motoneuron); CV6: Cerebral ventral 6 (motoneuron); CV7: Cerebral ventral 7 (motoneuron); N1M: N1 medial (central pattern generator interneuron); N1L: N1 Lateral (modulatory interneuron/central pattern generator interneuron); N2d: N2 dorsal (central pattern generator interneuron); N2v: N2 ventral (central pattern generator interneuron); N3p: N3 phasic (central pattern generator interneuron); N3t: N3 tonic (central pattern generator/modulatory interneuron); OC: Octopamine-containing interneuron (modulatory); OM: Esophageal mechanoreceptor; PlB: Pleural buccal neuron (inhibitory interneuron); PIR: Post-inhibitory rebound; SO: Slow oscillator (modulatory interneuron).

## Competing interests

The author declares that they have no competing interests.

## References

[B1] Marder E Calabrese RL Principles of rhythmic motor pattern generation Physiol Rev 1996 76 687 717 8757786 10.1152/physrev.1996.76.3.687

[B2] Selverston A Invertebrate central pattern generator circuits Phil Trans R Soc B 2010 365 2329 2345 10.1098/rstb.2009.0270 20603355 PMC2894947

[B3] Benjamin PR Elliott CJH Jacklet JW Snail feeding oscillator: the central pattern generator and its control by modulatory interneurons Neuronal and Cellular Oscillators 1989 New York, NY: Marcel Dekker 173 214

[B4] Staras K Kemenes I Benjamin PR Kemenes G Loss of self-inhibition is a cellular mechanism for episodic rhythmic behavior Curr Biol 2003 13 116 124 10.1016/S0960-9822(02)01435-5 12546784

[B5] Benjamin PR Roberts A, Roberts BL Gastropod feeding: behavioural and neural analysis of a complex multicomponent system Neural Origins of Rhythmic Movements. Symposia of the Society for Experimental Biology 1983 37 Cambridge, UK: Cambridge University Press 159 193 6679112

[B6] Kupfermann I A simple system for the study of motivation Behav Biol 1974 10 1 26 10.1016/S0091-6773(74)91644-7 4815142

[B7] Jing J Weiss KR Neural mechanisms of motor program switching in *Aplysia* J Neurosci 2001 21 7349 7362 11549745 10.1523/JNEUROSCI.21-18-07349.2001 PMC6762995

[B8] Jing J Weiss KR Generation of variants of a motor act in a modular and hierarchical motor network Curr Biol 2005 15 1712 1721 10.1016/j.cub.2005.08.051 16213817

[B9] Kristan W Gillette R North G, Greenspan RJ Behavioral choice Invertebrate Neurobiology 2007 Cold Spring Harbor, NY: Cold Spring Harbor Press 533 553

[B10] Kemenes G Staras K Benjamin PR Multiple types of control by identified interneurons in a sensory-activated rhythmic motor pattern J Neurosci 2001 27 2903 2911 11306642 10.1523/JNEUROSCI.21-08-02903.2001 PMC6762524

[B11] Kemenes I Straub VA Nikitin ES Staras K O'Shea M Kemenes G Benjamin PR Role of delayed synaptic plasticity in long-term associative memory Curr Biol 2006 16 1269 1279 10.1016/j.cub.2006.05.049 16824916

[B12] Straub VA Kemenes I O'Shea M Benjamin PR Associative memory stored by functional novel pathway rather than modification of pre-existing neuronal pathways J Neurosci 2006 26 4139 4146 10.1523/JNEUROSCI.0489-06.2006 16611831 PMC6673874

[B13] Marra V Kemenes I Vavoulis D Feng J O'Shea M Benjamin PR Role of tonic inhibition in associative reward conditioning in *Lymnaea* Front Behav Neurosci 2010 4 161 20877424 10.3389/fnbeh.2010.00161 PMC2944630

[B14] Kemenes I O'Shea M Benjamin PR Different circuit and monoamine mechanisms consolidate long-term memory in aversive and reward classical conditioning Eur J Neurosci 2011 33 143 152 10.1111/j.1460-9568.2010.07479.x 21070389

[B15] Straub VA Martinnen-Rossi E Styles BJ O'Shea M Benjamin PR Sensory activation of feeding and its relationship to appetitive conditioning in *Lymnaea* Soc Neurosci Abstract 2001 31 644.5

[B16] Yeoman MS Pieneman AW Ferguson GP ter Maat A Benjamin PR Modulatory role for the serotonergic cerebral giant cells in the feeding system of the snail *Lymnaea*. I. Fine wire recording in the intact animal and pharmacology J Neurophysiol 1994 72 1357 1371 7807217 10.1152/jn.1994.72.3.1357

[B17] Yeoman MS Brierley MJ Benjamin PR Central pattern generator interneurons are targets for the modulatory serotonergic cerebral giant cells in the feeding system of *Lymnaea* J Neurophysiol 1996 75 11 25 8822538 10.1152/jn.1996.75.1.11

[B18] Katz PS Hooper SL North G, Greenspan RJ Invertebrate central pattern generators Invertebrate Neurobiology 2007 Cold Spring Harbor, NY: Cold Spring Harbor Press 251 279

[B19] Kristan WB Neuronal decision-making circuits Curr Biol 2008 18 R928 R932 10.1016/j.cub.2008.07.081 18957243

[B20] Briggman KL Abarbanel HDI Kristan WB Optical imaging of neuronal populations during decision making Science 2005 307 896 901 10.1126/science.1103736 15705844

[B21] Rose RM Benjamin PR The relationship of the central motor pattern to the feeding cycle of *Lymnaea stagnalis* J Exp Biol 1979 80 137 163 501275 10.1242/jeb.80.1.137

[B22] Rose RM Benjamin PR Interneuronal control of feeding in the pond snail *Lymnaea *stagnalis II. The interneuronal mechanism generating feeding cycles J Exp Biol 1981 92 203 228

[B23] Benjamin PR Rose RM Central generation of bursting in the feeding system of the snail, *Lymnaea stagnalis* J Exp Biol 1979 80 93 118 227979 10.1242/jeb.80.1.93

[B24] Staras K Kemenes G Benjamin PR Pattern-generating role for motoneurons in a rhythmically active neuronal network J Neurosci 1998 18 3669 3688 9570798 10.1523/JNEUROSCI.18-10-03669.1998 PMC6793163

[B25] McCrohan CR Properties of ventral cerebral neurons in the feeding system of the snail, *Lymnaea stagnalis* J Exp Biol 1984 108 257 272

[B26] Elliott CJH Benjamin PR Interactions of pattern-generating interneurons controlling feeding in *Lymnaea stagnalis* J Neurophysiol 1985 54 1396 1411 4087040 10.1152/jn.1985.54.6.1396

[B27] Yeoman MS Parish DC Benjamin PR A cholinergic modulatory interneuron in the feeding system of the snail *Lymnaea* J Neurophysiol 1993 70 37 50 8103088 10.1152/jn.1993.70.1.37

[B28] Brierley MJ Staras K Benjamin PR Behavioral function of glutamatergic interneurons in the feeding system: plateauing properties and synaptic connections with motor neurons J Neurophysiol 1997 78 3386 3395 9405552 10.1152/jn.1997.78.6.3386

[B29] Brierley MJ Yeoman MS Benjamin PR Glutamate is the transmitter for the N2v retraction phase interneurons of the *Lymnaea *feeding system J Neurophysiol 1997 78 3408 3414 9405554 10.1152/jn.1997.78.6.3408

[B30] Straub VA Staras K Kemenes G Benjamin PR Endogenous and network properties of *Lymnaea *feeding central pattern generator interneurons J Neurophysiol 2002 88 1569 1583 12364488 10.1152/jn.2002.88.4.1569

[B31] Vehovszky A Elliott CJH Activation and reconfiguration of fictive feeding by the octopamine-containing modulatory OC interneurons in the snail *Lymnae* J Neurophysiol 2001 86 792 808 11495951 10.1152/jn.2001.86.2.792

[B32] Styles BJ Learning and sensory processing in a simple brain DPhil thesis 2004 University of Sussex, School of Life Sciences

[B33] Elliott CJH Benjamin PR Esophageal mechanoreceptors in the feeding system of the pond snail, *Lymnaea stagnalis* J Neurophysiol 1989 61 727 736 2723718 10.1152/jn.1989.61.4.727

[B34] Staras K Kemenes G Benjamin PR Electrophysiological and behavioural analysis of lip touch as a component of the food stimulus in the snail *Lymnaea* J Neurophysiol 1999 81 1261 1273 10085353 10.1152/jn.1999.81.3.1261

[B35] Straub VA Styles BJ Ireland JS O'Shea M Benjamin PR Central localization of plasticity involved in appetitive conditioning in *Lymnaea* Learn Mem 2004 11 787 793 10.1101/lm.77004 15537733 PMC534707

[B36] Elliott CJH Benjamin PR Interactions of the slow oscillator interneuron with the feeding pattern-generating interneurons in *Lymnaea stagnalis* J Neurophysiol 1985 54 1412 1421 4087041 10.1152/jn.1985.54.6.1412

[B37] Brierley MJ Yeoman MS Benjamin PR Glutamatergic N2 cells are central pattern generator interneurons of the *Lymnaea *feeding system: new model for rhythm generation J Neurophysiol 1997 78 3396 3407 9405553 10.1152/jn.1997.78.6.3396

[B38] Kemenes G Elliott CJH Analysis of feeding motor pattern in the pond snail, *Lymnaea stagnalis*: photoinactivation of axonally stained pattern generating interneurons J Neurosci 1994 14 153 166 8283231 10.1523/JNEUROSCI.14-01-00153.1994 PMC6576837

[B39] Vavoulis DV Straub VA Kemenes I Kemenes G Benjamin PR Dynamic control of a central pattern generator circuit: a computational model of the snail feeding network Eur J Neurosci 2007 25 2805 2818 10.1111/j.1460-9568.2007.05517.x 17561845

[B40] Kupfermann I Weiss KR The command neuron concept Behav Brain Sci 1978 1 3 39 10.1017/S0140525X00059057

[B41] McCrohan CR Initiation of feeding motor output by an identified interneuron in the snail *Lymnaea stagnalis* J Exp Biol 1984 113 351 366

[B42] McCrohan CR Kyriakides MA Cerebral interneurones controlling feeding motor output in the snail, *Lymnaea stagnalis* J Exp Biol 1989 147 361 374

[B43] Morgan PT Jing J Velim FS Weiss KR Interneuronal and peptidergic control of motor pattern switching in *Aplysia* J Neurophysiol 2001 87 49 61 11784729 10.1152/jn.00438.2001

[B44] Jing J Sweedler JV Cropper EC Alexeeva V Park J-H Romanova EV Xie F Dembrow NC Ludwar BC Weiss KR Vilim FS Feedforward compensation mediated by the central and peripheral actions of a single neuropeptide discovered using representational difference analysis J Neurosci 2010 30 16545 16558 10.1523/JNEUROSCI.4264-10.2010 21147994 PMC3072109

[B45] Elliott CJH Kemenes G Cholinergic interneurons inn the feeding system of the pond snail *Lymnaea stagnali*. II N1 interneurons make cholinergic synapses with feeding motoneurons Phil Trans R Soc B 1992 336 167 180 10.1098/rstb.1992.0054 1353265

[B46] Benjamin PR Staras K Kemenes G What roles do tonic inhibition and disinhibition play in the control of motor programs? Front Behav Neurosci 2010 4 30 20589095 10.3389/fnbeh.2010.00030 PMC2893002

[B47] McCrohan CR Benjamin PR Synaptic relationships of the cerebral giant cells with motoneurones in the feeding system of *Lymnaea stagnalis* J Exp Biol 1980 85 169 186 6246187 10.1242/jeb.85.1.169

[B48] Straub VA Benjamin PR Extrinsic modulation and motor pattern generation in a feeding network: a cellular study J Neurosci 2001 21 1767 1778 11222666 10.1523/JNEUROSCI.21-05-01767.2001 PMC6762967

[B49] Davis WJ Kovac MP Evarts EV, Wise SP, Bousfield D The command neuron and the organization of movement The Motor System in Neurobiology 1985 Amsterdam, The Netherlands: Elsevier Biomedical Press 73 80

[B50] Rose RM Benjamin PR Interneuronal control of feeding in the pond snail Lymnaea *stagnalis *I. Initiation of feeding cycles by a single buccal interneuron J Exp Biol 1981 92 187 201

[B51] Yeoman MS Vehovszky A Kemenes G Elliott CJH Benjamin PR Novel interneuron having hybrid modulatory-central pattern generator properties in the feeding system of the snail, *Lymnaea stagnalis* J Neurophysiol 1995 73 112 124 7714557 10.1152/jn.1995.73.1.112

[B52] Katz PS Neuromodulation intrinsic to the central pattern generator for escape swimming in *Tritonia* Ann NY Acad Sci 1998 860 181 188 10.1111/j.1749-6632.1998.tb09048.x 9928311

[B53] Vehovszky A Szabo H Elliott CJH Octopamine-containing (OC) interneurons enhance central pattern generator activity in sucrose-induced feeding in the snail *Lymnaea* J Comp Physiol A 2004 190 837 846 10.1007/s00359-004-0539-y 15316729

[B54] Elliott CJH Verhovszky A Polycyclic neuromodulation of the feeding rhythm of the pond snail *Lymnaea stagnalis *by the intrinsic octopaminergic interneuron, OC Brain Res 2000 887 63 69 10.1016/S0006-8993(00)02968-1 11134590

[B55] Vehovszky A Elliott CJH Heterosynaptic modulation by the octopaminergic OC interneurons increases the synaptic outputs of protraction phase interneurons (SO, N1L) in the feeding system of *Lymnaea stagnalis* Neurosci 2002 115 483 494 10.1016/S0306-4522(02)00414-1 12421615

[B56] Vehovszky A Elliott CJH Octopamine increases the excitability of neurons in the snail feeding system by modulation of inward sodium current but not outward potassium currents BMC Neurosci 2005 6 70 10.1186/1471-2202-6-70 16332252 PMC1351263

[B57] Tuersley MD How is food arousal manifested in the pond snail *Lymnaea stagnalis*? An overview J Mollusc Stud 1989 55 209 216 10.1093/mollus/55.2.209

[B58] Whelan HA McCrohan CR Effect of satiation on synaptic input to an identified interneurone in the isolated nervous system of *Lymnaea stagnalis* J Physiol 1993 467 1275P

[B59] Alania M Sakharov DA Elliott CJH Multilevel inhibition of feeding by a peptidergic pleural interneuron in the mollusc *Lymnaea stagnalis* J Comp Physiol A 2004 190 379 390 10.1007/s00359-004-0503-x 15042400

